# P-854. Designing and Implementing a Remote Prospective Audit-and-Feedback Intervention in Nursing Homes

**DOI:** 10.1093/ofid/ofaf695.1062

**Published:** 2026-01-11

**Authors:** Sally Jolles, Grace Multhauf, Shivani Patel, Janae Adams, Jon P Furuno, Emily K Short, Chandler C Nissen, Veronica Mateo, Kendall J Tucker, YoungYoon Ham, Christopher J Crnich

**Affiliations:** University of Wisconsin School of Medicine and Public Health, Madison, WI; University of Wisconsin School of Medicine and Public Health, Madison, WI; Mount Carmel Health System, Columbus, Ohio; University of Wisconsin-Madison, Madison, Wisconsin; Oregon State University, Portland, Oregon; Oregon State University College of Pharmacy, Portland, Oregon; Oregon State University, Portland, Oregon; Oregon State University, Portland, Oregon; Oregon State University College of Pharmacy, Portland, Oregon; Oregon Health & Science University, Portland, Oregon; University of Wisconsin School of Medicine and Public Health, Madison, WI

## Abstract

**Background:**

The optimal approach to antibiotic stewardship in nursing homes (NHs) remains poorly understood. Prospective audit-and-feedback (PAF) is a highly effective antibiotic stewardship strategy typically employed in hospitals with robust pharmacy and information system resources. It is unclear if PAF can be effectively performed in lower resource settings like NHs.

**Figure 1:**
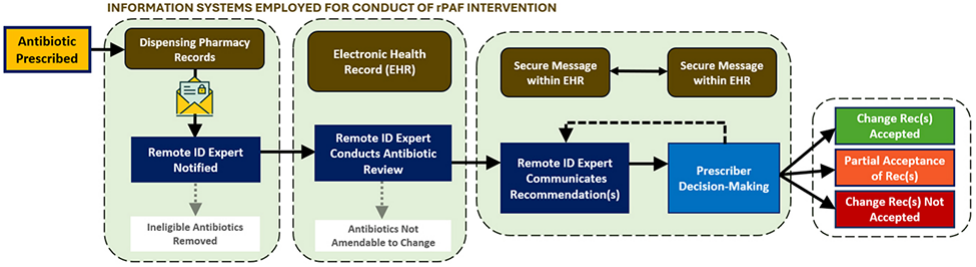
rPAF Pilot Workflow

**Table 1:**
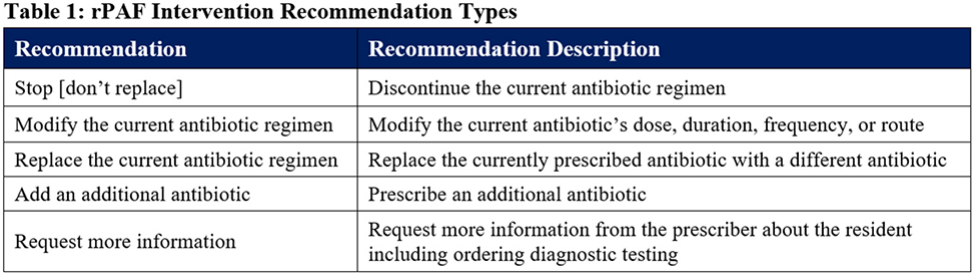
rPAF Intervention Recommendation Types

**Methods:**

We conducted a multi-phase mixed-methods study to design and implemented a remote PAF (rPAF) intervention in a NH network in the Northwest United States. We conducted interviews with key informants and performed document analyses, guided by the Systems Engineering Initiative for Patient Safety (SEIPS) model, to design a beta-version of the rPAF intervention. Information generated from cognitive interviews structured around three clinical scenarios and presented to NH providers in the NH EHR training environment was used to refine the rPAF intervention. A REDCap database was constructed to enter prospective data on antibiotic regimens reviewed, recommendation acceptance, and clinical characteristics and outcomes of residents.

**Results:**

A final workflow involving information sharing between the NH dispensing pharmacy and a remotely located infectious disease (ID) pharmacist who, in turn conveyed antibiotic regimen change recommendations to NH providers via encrypted EHR message was developed (Figure 1). Key informant interviews and document analyses identified a need to limit the size and content of encrypted messages and include concrete justifications for the recommendations provided. ID pharmacists reviewed 58 antibiotic regimens in 13 study NHs during the first three weeks of the four-month pilot study and made 21 antibiotic recommendations. These recommendations included 10 modifications, 5 stops, 4 replacements, and 2 requests for more information (see Table 1 for explanation of types).

**Conclusion:**

Our initial findings demonstrate the feasibility of PAF in NHs, based on using remote ID pharmacist review and encrypted EHR messaging. Evaluation of the rPAF on antibiotic prescribing is ongoing and will also include assessment of the costs and sustainability of the rPAF intervention as well as the scalability of the rPAF intervention.

**Disclosures:**

Sally Jolles, MA, MS, Merck: Grant/Research Support Jon P. Furuno, PhD, Merck: Grant/Research Support Kendall J. Tucker, PharmD, MS, Merck: Grant/Research Support Christopher J. Crnich, MD, PhD, Merck: Grant/Research Support

